# Genome Evolution in the Primary Endosymbiont of Whiteflies Sheds Light on Their Divergence

**DOI:** 10.1093/gbe/evv038

**Published:** 2015-02-25

**Authors:** Diego Santos-Garcia, Carlos Vargas-Chavez, Andrés Moya, Amparo Latorre, Francisco J. Silva

**Affiliations:** ^1^Institut Cavanilles de Biodiversitat i Biologia Evolutiva, Universitat de València, Spain; ^2^Unidad Mixta de Investigación en Genómica y Salud, FISABIO-Salud Pública and Universitat de València, Spain

**Keywords:** *Portiera*, amino acid biosynthesis, endosymbiont, genome stasis, genome reduction, molecular evolution, divergence time, whiteflies

## Abstract

Whiteflies are important agricultural insect pests, whose evolutionary success is related to a long-term association with a bacterial endosymbiont, *Candidatus Portiera aleyrodidarum*. To completely characterize this endosymbiont clade, we sequenced the genomes of three new *Portiera* strains covering the two extant whitefly subfamilies. Using endosymbiont and mitochondrial sequences we estimated the divergence dates in the clade and used these values to understand the molecular evolution of the endosymbiont coding sequences. *Portiera* genomes were maintained almost completely stable in gene order and gene content during more than 125 Myr of evolution, except in the *Bemisia tabaci* lineage. The ancestor had already lost the genetic information transfer autonomy but was able to participate in the synthesis of all essential amino acids and carotenoids. The time of divergence of the *B. tabaci* complex was much more recent than previous estimations. The recent divergence of biotypes B (MEAM1 species) and Q (MED species) suggests that they still could be considered strains of the same species. We have estimated the rates of evolution of *Portiera* genes, synonymous and nonsynonymous, and have detected significant differences among-lineages, with most *Portiera* lineages evolving very slowly. Although the nonsynonymous rates were much smaller than the synonymous, the genomic d*N*/d*S* ratios were similar, discarding selection as the driver of among-lineage variation. We suggest variation in mutation rate and generation time as the responsible factors. In conclusion, the slow evolutionary rates of *Portiera* may have contributed to its long-term association with whiteflies, avoiding its replacement by a novel and more efficient endosymbiont.

## Introduction

Whiteflies (Hemiptera: Sternorrhyncha: Aleyrodidae) are a family of hemimetabolous insects, which, like other hemipterans, are plant sap suckers. Their diets are unbalanced with a high content of carbohydrates but a low content of the amino acids essential for insects (Douglas 1998; Baumann 2005). One strategy to fulfill their nutritional requirements has been the establishment of different symbiotic associations, including endosymbiosis, with a wide range of microorganisms. All whiteflies have a paired bacteriome that is usually orange in color (Buchner 1965). It is composed by specialized cells called bacteriocytes, which always present a pleomorphic bacterium, *Candidatus* (*Ca.*) *Portiera aleyrodidarum* (hereafter *Portiera*) (Thao and Baumann 2004). *Portiera* is an obligate primary endosymbiont located in host-derived vesicles and displaying a typical three-membrane system with one membrane derived from the insect vacuole (Santos-Garcia, Silva et al. 2014). It belongs to family Halomonadaceae and, with the endosymbionts of psyllids (*Ca. Carsonella ruddii*, hereafter *Carsonella*) and moss bugs (*Ca. Evansia muelleri*, hereafter *Evansia*), it forms a phylogenetic clade currently composed exclusively by hemipteran endosymbionts (Kuechler et al. 2013; Santos-Garcia et al. 2014). The concordance of their phylogeny with the one of their hosts, and several other endosymbiont genomic features have led to the proposal that the start of the endosymbiotic event took place in the ancestor of psyllids and whiteflies (Santos-Garcia et al. 2014), both considered to be included in the lineage Psylliformes or Psyllinea (Shcherbakov 2000; Drohojowska and Szwedo 2015). After this event, insects and endosymbionts coevolved leading to the obligate relationships psyllids/*Carsonella* on one hand and whiteflies/*Portiera* on the other. This event should have taken place in, or before, the Early Jurassic (201–174 Ma), based on the oldest Psylloidea fossil (Ouvrard et al. 2010). In addition, whiteflies may harbor several facultative endosymbionts that share the bacteriocyte with *Portiera* (Gottlieb et al. 2008). However, the potential benefits of these endosymbionts are not yet clear.

The family Aleyrodidae is formed by four subfamilies, although the taxonomic status of only three of them is unquestionably recognized (Drohojowska and Szwedo 2015). They are the extant subfamilies Aleyrodinae and Aleurodicinae and the extinct subfamily Bernaeinae. The oldest fossil registry of a whitefly (Bernaeinae) can be traced to the Late Jurassic, whereas the oldest Aleyrodinae (*Baetylus kahramanus*) and Aleurodicinae (*Gapenus rhinariatus*) fossils are dated at the Early Cretaceous (approximately 135–125 Ma) (Campbell et al. 1994; Drohojowska and Szwedo 2011, 2013, 2015). The most relevant extant species in the subfamily Aleyrodinae is *Bemisia tabaci*, which is an important agricultural pest. Its taxonomic status is controversial, and while in early works, it was classified in biotypes, now it is considered a complex of morphologically indistinguishable species clustered in 11 well-defined high-level groups (De Barro et al. 2011). Two of these species/biotypes were *B. tabaci* MEAM1 (biotype B) and *B. tabaci* MED (biotype Q), whose divergence was recently estimated at 13 Myr (Boykin et al. 2013), a value that disagrees with the high nucleotide identity of the genes of their *Portiera* strains (Santos-Garcia et al. 2012; Sloan and Moran 2012a).

To date, five genomes of *Portiera* have been sequenced. Four are endosymbionts of *B. tabaci* (Santos-Garcia et al. 2012; Sloan and Moran 2012a; Jiang et al. 2013) and one of *Trialeurodes vaporariorum* (Sloan and Moran 2013). These whiteflies belong to the subfamily Aleyrodinae and their endosymbionts presented extremely reduced genomes (less than 400 kb) encoding for different functions involved in the synthesis of amino acids and carotenoids, which are important to complement their hosts diets. However, *Portiera* from *B. tabaci* shows some relevant features, very unusual in primary endosymbionts, such as low coding density, large intergenic regions, and a high number of tandem repeats. When the *B. tabaci* lineage was compared with the one from *T. vaporariorum*, less genes were detected in the former in spite of its larger genome (approximately 80 kb) (Sloan and Moran 2013). Important differences in genome size among strains of an obligate endosymbiont species have been already reported for a limited number of species, the most relevant being the primary endosymbiont of aphids (*Buchnera aphidicola*) (Shigenobu et al. 2000; Gil et al. 2002; Tamas et al. 2002; van Ham et al. 2003; Pérez-Brocal et al. 2006; Moran et al. 2009; Lamelas et al. 2011) and carpenter ants (*Ca. Blochmannia* spp.) (Gil et al. 2003; Degnan et al. 2005; Williams and Wernegreen 2010, 2013).

**Fig. 1.—:**
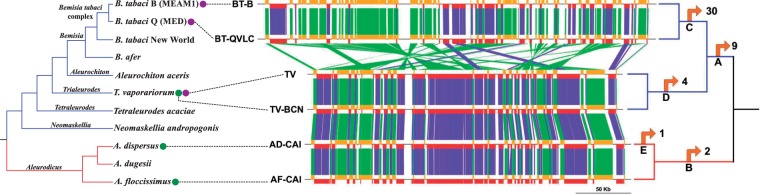
Simplified cladrogram showing different whiteflies species (left) and genomic synteny in *Portiera* strains (right). Whiteflies subfamilies are represented by colored branches, blue for Aleyrodinae and red for Aleurodicinae. Genera are displayed as branch labels. Green dots denote the *Portiera* genomes reported in this work. Purple dots denote already sequenced *Portiera* genomes. Orange boxes represent syntenic genes in the direct strand, red boxes genes in the complementary strand, green lines connect genes with at least one of them in the direct strand whereas blue lines connect genes when both are in the complementary strand. The cladogram on the right represents the different host subfamilies (same as above) and the gene losses in each branch represented by a letter (listed in table 2).

In this work we have sequenced the genomes of three additional *Portiera* strains, two of them belonging to Aleurodicinae (*Aleurodicus dispersus* and *Aleurodicus floccissimus*) and the other to Aleyrodinae (*T. vaporariorum*), with the aim of comparing their genomic features, reconstructing their last common symbiont ancestor and determining the genome evolution in the different whiteflies subfamilies lineages (fig. 1). We have also estimated the divergence dates among them and used these values to understand the molecular evolution of their coding sequences (CDS).

## Materials and Methods

### Insect Samples, Genome Amplification, and Sequencing

*Trialeurodes vaporariorum* TVAW-BCN was a field population collected in Barcelona (Spain), whereas *A. dispersus* ADAW-CAI and *A. floccissimus* AFAW-CAI samples were collected from field populations in the Canary Islands (Spain). The three samples contained two secondary endosymbionts (*Arsenophonus* sp. and *Wolbachia* sp.). Single bacteriomes were extracted from fourth-instar larvae (red eyes) using glass capillaries and used for Whole Genome Amplification (GenomiPhi V2, GE Healthcare). Each bacteriome was transferred to 0.2-ml polymerase chain reaction tubes containing 10 μl of fresh made lysis solution (400 mM KOH, 10 mM ethylenediaminetetraacetic acid, 100 mM Dithiothreitol) and left 10 min on ice. Lysis solution was neutralized with fresh made neutralization buffer (400 mM HCl, 600 mM Tris–HCl pH 7.5) and reaction mix was added (7 μl Sample Buffer, 9 μl Reaction Buffer, and 1 μl Enzyme Mix). Amplification reaction profile was: 30°C for 90 min and 65°C for 10 min. For each species, ten reactions (ten bacteriomes from different individuals) were made and pooled to diminish the impact of the potential chimeras formed during Whole Genome Amplification. Pooled samples were sequenced using Roche 454 GS-FLX Titanium single-end (700 bp length in average) and an Illumina HiSeq 2000 MPET (3 kb insert size).

### Genome Assembly and Annotation

For a detailed description of this section, see the supplementary material and methods, Supplementary Material online.

### Phylogenetic Relationships

Mitochondrial cytochrome c oxidase subunit 1 (*COI*) sequences from *B. tabaci*, *T. vaporariorum,* and *Aleurodicus dugesii* (Thao et al. 2004) were used for read identification in the species of this study and for the *B. tabaci* QHC-VLC strain (Santos-Garcia et al. 2012). MIRA v4.0 assembler (EST mode) (Chevreux et al. 1999) was used for assembly of the selected reads and an iterative mapping and assembly approach was followed for obtaining *COI* gene sequences: *T. vaporariorum* TVAW-BCN (LN614547), *A. dispersus* ADAW-CAI (LN614548), *A. floccissimus* AFAW-CAI (LN614549), and *B. tabaci* QHC-VLC (LN614545). A *COI* sequence of a *B. tabaci* B (MEAM1) (LN614546) laboratory strain from Israel was amplified with the universal primer LCO1490 (Folmer et al. 1994) combined with the L2-N-3014 primer (Khasdan et al. 2007) and sequenced by Sanger.

Different available whiteflies *COI* sequences were downloaded from National Center for Biotechnology Information (NCBI) nucleotide database and aligned against assembled COI sequences with MAFFT (L-INS-i algorithm) (Katoh et al. 2002). Two data sets were generated due to the different sequence lengths (corresponding with the 5′- and 3′-region of the *COI* gene) and alignments were refined with Gblocks (Castresana 2000). jModeltest2 (Darriba et al. 2012) was used for selecting the best model for each data set based on Akaike Information Criterion. In both data sets, MtArt plus gamma distribution (MtArt + G) was the best model. Maximum-likelihood (ML) trees were generated using RaxML with optimizations for branch lengths and model and 500 rapid bootstrap replicates (Stamatakis 2006). Generated ML trees were used as starting tree for a Bayesian phylogenetic inference with PhyloBayes3, under the MtArt +G model, and allowing the convergence of the chains (all effective sample sizes, ESS, were above 200) (Lartillot et al. 2009). *Acyrthosiphon pisum COI* gene was used as outgroup. Tree visualization and editing were performed with Archaeopterix (Han and Zmasek 2009).

### Comparative Genomics and Genome Stasis

Proteomes from the newly reported *Portiera* (TV-BCN, AD-CAI, and AF-CAI) plus the ones already published BT-QVLC (CP003835), BT-B (CP003708), and TV (CP004358) (Santos-Garcia et al. 2012; Sloan and Moran 2012a, 2013) were used as input for OrthoMCL (1.5 inflation value, 70% match cutoff, 1 x 10^−^^5^ *e* value cutoff) (Li et al. 2003; Manzano-Marín et al. 2012). Cluster of orthologous groups of proteins (COG) categories were assigned to each orthologous cluster with a custom perl script (Tatusov et al. 2003). Genome synteny between *Portiera* strains was plotted using genoPlotR package (Guy et al. 2010) from R software (R Core Team 2013). MGR was used for genome rearrangement inference (Bourque and Pevzner 2002).

### Divergence Time of *Portiera* Lineages

Two data sets were collected for dating the divergence between the different *Portiera* strains: BT-QVLC, BT-B, TV, TV-BCN, AD-CAI, AF-CAI, and the free-living relatives *Halomonas elongata* and *Chromohalobacter salexigens* (Copeland et al. 2011; Schwibbert et al. 2011; Santos-Garcia et al. 2012; Sloan and Moran 2012, 2013). The A data set was composed of *rpoB*, *rpoC*, *carB* and *dnaE* and the B data set of *sucA*, *aceE*, *valS* and *leuS* genes. All these genes were in the top of the longest genes found in *Portiera* genomes. A third data set, composed of some whiteflies *COI* gene sequences, was collected from a previous work (Thao et al. 2004) and from this work: *B. tabaci* MED QHC-VLC (LN614545), MEAM1 (LN614546) and New World (AY521259), *T. vaporariorum* TV (AY521265) and TVAW-BCN (LN614547), *A. dugesii* (AY521251), *A. dispersus* ADAW-CAI (LN614548), *A. floccissimus* AFAW-CAI (LN614549), *Aleurochiton aceris* (AY572538), *Neomaskellia andropogonis* (AY572539), and *Tetraleurodes acaciae* (AY521262). *Acyrthosiphon pisum* (FJ411411) was used as outgroup.

Codon-based alignments for each gene were obtained by aligning the translated protein with MAFFT (L-INS-i algorithm) (Katoh et al. 2002) and back-translating with PAL2NAL (Suyama et al. 2006). Alignments were refined with Gblocks (Castresana 2000) and the best evolutionary model was selected with jModeltest2 (Darriba et al. 2012). Substitution saturation was checked for each gene alignment, according to its evolutionary model and its partition scheme, with Xia’s method implemented in DAMBE5 (Xia et al. 2003; Xia 2013).

Divergence estimation was first computed with BEAST v2.0.2 (Bouckaert et al. 2014). BEAUti was used to process the alignments and build the xml files. For each gene, the evolutionary model was selected with jModeltest2 and used as priors. Although the *Portiera* data sets were not partitioned for avoiding the increase in the model complexity, the *COI* data set was partitioned into codon positions (1+2 and 3). A lognormal relaxed clock with a Yule speciation process was selected for all data sets based on the results of the model comparison plugin (harmonic mean of the posterior probabilities with 100 bootstrap) implemented in Tracer v1.6 (Rambaut 2007). Two calibration points were inferred from previous works and set to a uniform distribution. They were the emergence of the Sternorrhyncha suborder (278-250 Ma) and the divergence between the subfamilies Aleyrodinae and Aleurodicinae (135–125 Ma) (Wootton 1981; Shcherbakov 2000; Grimaldi and Engel 2005; Drohojowska and Szwedo 2011, 2013, 2015; Shi et al. 2012). One calibration point was used in the *Portiera* data sets, whereas two calibration points were used in the *COI* data sets. Each data set was first run under the prior to ensure that divergence dates are only estimated from the data and are not produced by the selected priors. Finally, eight independent runs were performed allowing 500 million generations and sampling every 50,000th generation. Convergence, ESS suitability (larger than 200), and burn-in of the runs were checked and calculated with Tracer v1.6. Log files of the convergent runs were trimmed, reduced, and combined with Logcombiner and used for obtaining the descriptive statistics with Tracer v1.6. For obtaining an averaged value of *Portiera* divergence for downstream analyses, both data set (A and B) were used in conjunction as BEAST v2.0.2 input as explained above. Tree topologies were obtained with TreeAnnotator and FigTree v1.3.1 (Rambaut 2007).

To ensure the robustness of the obtained dates, PhyloBayes3 was used for dating the divergences with the same data sets (Lartillot et al. 2009). Because PhyloBayes3 does not accept gene or codon partition, each *Portiera* data set alignment was concatenated and the *COI* data set was run without codon partition. Because fixed tree topologies are required for Phylobayes3, the topologies obtained from BEAST analyses were used as input. Evolutionary models were selected as explained above and a chain under the prior was run for each data set. Finally, three independent chains were run for each data set until discrepancy between chains was less than 0.1 and ESS were above 200 (Lartillot et al. 2009). Descriptive statistics were obtained with the readdiv script from PhyloBayes3.

### Molecular Evolution in *Portiera* and Mitochondria

All orthologous protein clusters shared between *Portiera* strains BT-QVLC, TV-BCN, AD-CAI, and AF-CAI (240 proteins) were aligned with MAFFT (L-INS-i algorithm) (Katoh et al. 2002). Codon-based alignments were obtained as explained above. Codeml from PAML package (Yang 2007) was used to obtain the d*S* and d*N* values of each gene. Three branch models were used: m0 (one *ω*), m1 (free *ω* ratios in each branch), and m2 (2 *ω* with *Portiera* from *B. tabaci* as foreground branch). The best model for each orthologous cluster was selected using the likelihood ratio test values and the chi2 tool from PAML.

Statistical analyses were performed with R (R Core Team 2013). Substitution rates per year were calculated based on the results from the divergence dates estimated for each *Portiera* lineage. Exploratory analyses (descriptive statistics, histograms and density plots, boxplots, etc.) were used for cleaning the data of outliers and zero values (probably produced by decimal limits in codeML). Levene’s test (homoscedasticity) and Shaphiro’s test (normality) were used as a previous step to select the appropriate statistical test. After logarithmic transformation (base 10) most of the distributions fitted a normal distribution but some of them presented unequal variances. Two types of tests were used to check statistical differences between d*N*, d*S*, or *ω* distributions among *Portiera* strains. The Student’s *t*-test for equal and unequal (Welch’s procedure) variances was used when the data fitted a normal distribution. Kruskal–Wallis test, with its corresponding post hoc tests with *P* values corrected by Bonferroni’s procedure, was used when the data were not normally distributed but presented equal variances. A statistical significance (*α*) of 0.01 was used for all the statistical tests. Finally, substitution rates at genomic level were calculated as a weighted arithmetic mean of all the genes used.

Codon-based alignment of *COI* sequences from *B. tabaci* QHC-VLC, *T. vaporariorum* TVAW-BCN, *A. dispersus* ADAW-CAI, and *A. floccissimus* AFAW-CAI was performed with RevTrans (Wernersson and Pedersen 2003) and refined with Gbloks (Castresana 2000) (1,341-bp final alignment). d*S* and d*N* values were obtained with codeml as explained above.

## Results

### Genomic Features of *Portiera* Strains

The genomes of *Portiera* strains TV-BCN, AD-CAI, and AF-CAI are composed of a single circular chromosome with an approximate average coverage for each genome of 90× and 1,500× for 454 and Illumina libraries, respectively. The general features of the three new *Portiera* genomes are roughly similar to those of the previously sequenced *Portiera* genomes and to their sister Halomonadaceae lineages (table 1) (Santos-Garcia, Latorre et al. 2014). They have extremely reduced genomes (between 280 and 290 kb) with low GC contents and high coding densities but they do not display the large intergenic regions observed in *Portiera* from *B. tabaci* (only *Portiera* BT-QVLC is shown in table 1 and supplementary fig. S1, Supplementary Material online) (Santos-Garcia et al. 2012; Sloan and Moran 2012a, 2013).

**Table 1:** General Genomic Features of *Portiera* Strains and Related Endosymbionts

Symbiont	*Carsonella* HC	*Portiera* TV	*Portiera* TV-BCN^a^	*Portiera* AD-CAI^a^	*Portiera* AF-CAI^a^	*Portiera* BT-B	*Portiera* BT-QVLC^b^	*Evansia* Xc1
Host	Hcu	Tva	Tva	Adi	Afl	Bta B	Bta Q	Xca
Genome size (bp)	166,163	280,663	280,822	290,195	290,376	358,242	357,472	357,498
GC%	14	25	25	24	24	26	26	25
Genes	223	307	307	318	317	292	284	369
CDS	192	269	268	279	278	256	247	330
Coding density (%)	98	94	94	95	95	69	68	94
rRNA	3	3	3	3	3	3	3	3
tRNA	28	34	34	34	34	33	33	33
Other RNA	0	1	2	2	2	0	2	3
Pseudo	0	0	1	1	0	3	7	0

Note.—Hcu, *Heteropsylla cubana*; Tva, *Trialeurodes vaporariorum*; Adi, *Aleurodicus dispersus*; Afl, *Aleurodicus floccissimus*; Bta, *Bemisia tabaci*; Xca, *Xenophyes cascus*.

^a^This work.

^b^Re-annotated for this work.

The three new *Portiera* strains contain 39 noncoding RNA genes, which specify 34 tRNAs able to decode all mRNAs, the three rRNAs (*16S*, *23S,* and *5S*), one transfer-messenger RNA (tmRNA), and the RNA subunit of RNase P (*rnpB*). The size differences among the three new genomes are of only 10 kb (ten coding genes). The three new genomes maintain a clear GC skew pattern, which is not appreciable in any of the sequenced *Portiera* strains from *B. tabaci*. Furthermore, although all *Portiera* genomes have inverted and tandem repeats, it seems that they were mainly accumulated in the Aleyrodinae endosymbionts and specifically in *B. tabaci* lineage (supplementary fig. S1, Supplementary Material online). The sequence of *Portiera* TV-BCN was almost 100% identical to the one of the previously sequenced TV strains (Sloan and Moran 2013). The only differences in genome annotation are due to the annotations of the tmRNA gene and of *miaA* as a pseudogene in TV-BCN.

### Comparative Genomics and Genome Stasis in *Portiera*

Proteomes from the three new *Portiera* strains plus BT-QVLC were used to infer the pangenome and the core genome of *Portiera* (fig. 1). Four hypothetical proteins without significant similarity beyond *Portiera* BT proteomes were not included in the analysis. The bifunctional protein encoded by *alaS* was included as two different proteins due to its presence as *alaXp* in *Portiera* from *Bemisia* and *Trialeurodes* lineages, the gene fission in *Portiera* from *A. dispersus* (*alaS* plus *alaXp*), and the full gene present in *Portiera* from *A. floccissimus*. The core genome and the pangenome are composed of 240 and 280 proteins, respectively (supplementary fig. S2, Supplementary Material online).

Most of these differences were due to the presence of *Portiera* BT-QVLC; had it not been included, only 12 genes would be absent of the core: *lepB* only carried by Portiera TV-BCN, *ahpC* that is shared by AF-CAI and BT-QVLC and 11 genes shared by AD-CAI and AF-CAI (two of them also shared with BT-QVLC) (supplementary fig. S2 and tables S1 and S2, Supplementary Material online). This suggests that the Last Common Ancestor (LCA) of all *Portiera* strains already possessed an extremely reduced genome with 280 coding genes (considering *alaS* as a single gene and the ortholog of *PAQ_201*, only present in *B. tabaci* strains, as pseudogene). Proteins were assigned to COG categories. Categories J (translation) and E (amino acid metabolism) were those with the highest numbers of hits (supplementary fig. S3, Supplementary Material online). The largest among-strain difference was observed in the L (replication, recombination, and repair) category.

Gene order comparison showed that all *Portiera* genomes, regardless of belonging to Aleyrodinae or to Aleurodicinae, were syntenic except those from the lineage leading to *Portiera* strains from *B. tabaci* (fig. 1). When gene (coding and noncoding) losses were ascribed to phylogenetic branches, we observed that the majority of gene losses took place in branches C and A (fig. 1 and table 2) and that the genome of *Portiera* AF-CAI resembles, both in gene order and in gene content, the ancestral *Portiera* genome.

**Table 2:** Gene Losses during *Portiera* Evolution

	Branch				
	A	B	C	D	E
Gene losses	*miaA*,^a^ *rnc*,^a^ *rpmD*,^a^ *glyA,*^c^ *alaS*,^a^ *hupB*,^a^ *tktA, metG*,^a^ *yqgF*^a^	*lepB,*^d^ *PAQ_201*	*dnaQ*,^b^ *dnaX*,^b^ *dnaN*,^b^ *holA*,^b^ *holB*,^b^ *ruvC*,^b^ *ssb*,^b^ *mutL*,^b^ *upp, clpP,*^d^ *clpX,*^d^ *clpB,*^d^ *lspA,*^d^ *sohB,*^d^ *lepB,*^c^ *mucD, dapB*,^b^ *lysA,*^c^ *argH,*^c^ *dapF,*^c^ *trpS*,^a^ *rsmA*,^a^ *frr*,^a^ *deaD*,^a^ *tRNA-Ala*,^a^ *era, lipB, galP, PAQ_201*	*hisE,*^c^ *ahpC, rplA*,^a^ *PAQ_201*	*ahpC*

^a^Transcription, translation, and ribosome biogenesis.

^b^Replication, recombination, and repair.

^c^Amino acid biosynthesis.

^d^Posttranslational modification, protein turnover, and chaperones.

### Metabolic Blueprint of *Portiera* Strains

**Fig. 2.—:**
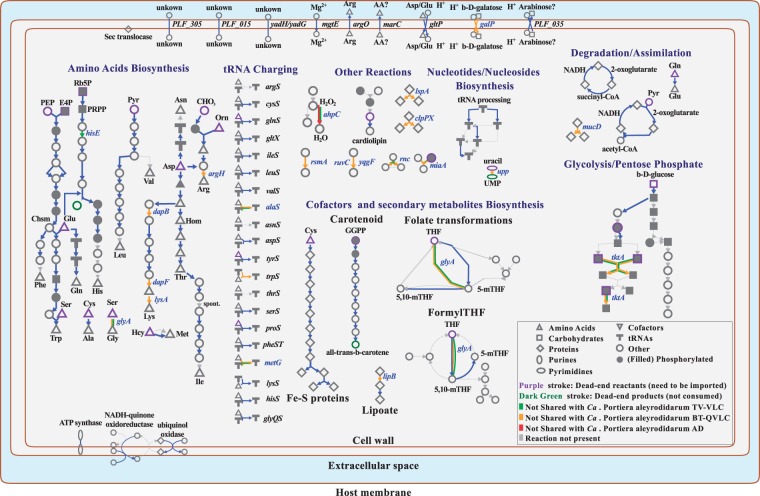
Metabolic comparison of *Portiera* strains. Four strains were analyzed in this work. Metabolism from *Portiea* AF-CAI was assumed as the basal one (shared between all strains) and is represented by blue lines and arrows. Arrows indicate the direction of the reaction. Gene losses from different strains are displayed in a representative color. Gene names in blue denote pseudogenes at least in one strain. Chsm, chorismate; GGPP, geranylgeranyl diphosphate; THF, tetrahydrofolate; Pyr, pyruvate; PEP, phosphoenolpyruvate; PRPP, 5-phospho-a-d-ribose-1-diphosphate; Hcy, homocysteine.

The ancestral *Portiera* metabolism has been maintained basically unchanged during its evolution in Aleurodicinae, whereas some gene losses took place in Aleyrodinae, especially in the *B. tabaci* lineage. *Portiera* AF-CAI, which has the most complete metabolism, was used as a reference for comparing the metabolism of the other different *Portiera* strains (blue arrows in fig. 2). All the strains can produce carotenes, the Fe–S cluster proteins, decarboxylate pyruvate for producing some intermediate metabolites and reducing power (NADH) to maintain most of the aerobic electronic transporter chains (*nuo* operon and ubiquinol oxidase) and the ATP synthase.

Most of the metabolism is devoted to the biosynthesis of amino acids. Lysine, arginine, threonine, tryptophan, and glycine are synthesized within *Portiera* cells, whereas for the synthesis of phenylalanine, isoleucine, valine, leucine and histidine, the complementary support of external enzymes (probably from the host) is required to complete the pathways. In addition, although it does not encode a complete methionine pathway, it has retained *metE*, the gene controlling the last step of the pathway. The substrate of this reaction, homocysteine, is probably obtained from the host. In contrast, *Portiera* strains from Aleyrodinae (especially *B. tabaci* strains) show a less complete amino acid metabolism. Both BT-QVLC and TV-BCN have lost *tktA*, one gene involved in the pentose phosphate pathway and in the production of d-erythrose 4-phosphate and d-ribulose-5-phosphate. These compounds are linked with the synthesis of histidine, phenylalanine, and tryptophan (fig. 2). Also, they have lost *glyA* and, thus, the ability to synthesize glycine and make folate transformations. Additionally, TV-BCN and BT-QVLC have lost genes for the synthesis of arginine and histidine, and arginine and lysine, respectively.

Although the genomes of all *Portiera* strains contain sets of tRNA genes for all amino acids, the ability for tRNA aminoacylation is incomplete. The genes *argS* and *thrS* are absent in all *Portiera* genomes. Although the gene (*asnS*) is also absent, the synthesis of Asn-tRNA^Asn^ may be performed by the alternative pathway encoded by *aspS* (a nondiscriminant enzyme between tRNA^Asp^ and tRNA^Asn^) and *gatABC* (Bernard et al. 2006). Three more genes encoding aminoacyl tRNA synthetases have been lost in *Portiera* BT-QVLC (*alaS*, *metG,* and *trpS*). The first two were also lost in *Portiera* TV-BCN. The gene *alaS* in *Portiera* AF-CAI, as in other bacteria, encodes a bifunctional protein composed of the aminoacylation domain (amino end) and two C-terminal domains, one of them responsible for editing the miss-charged tRNA^Ala^, to avoid their lethal effects (Guo et al. 2009). *Portiera* AD-CAI encodes both domains in separate genes (*alaS* and *alaXp*), whereas in BT-QVLC and TV-BCN, only *alaXp* was maintained. Regarding replication, recombination and repair, the only genome with relevant differences was that of *Portiera* BT-QVLC. Like other *Portiera* strains from *B. tabaci* (Sloan and Moran 2012a), it has lost up to nine genes, including some encoding DNA polymerase III subunits. From the ten transporters probably present in the *Portiera* LCA, the galactose (*galP*) is a pseudogene in BT-QVLC, suggesting that different sugar molecules may pass through diffusion across the membranes. Although few of these transporters have a known ligand, most of them should have a wide range of targets because all *Portiera* strains need to import mostly the same compounds/amino acids (see purple strokes in fig. 2) and not all of them can pass freely across membranes.

### Divergence Times of *Portiera* Lineages

**Fig. 3.—:**
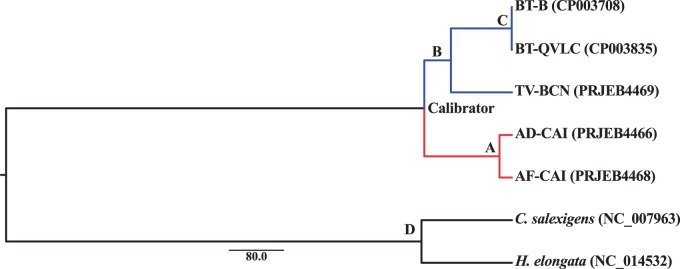
BEAST2 Bayesian inferred tree of *Portiera* strains. Each node whose divergence time was estimated is denoted by a bold uppercase letter (see table 3). Each strain is displayed with its accession number, or project number, in brackets. All posterior probabilities were 1. Branch lengths are displayed in Myr. *Chromohalobacter salexigens* and *H. elongata* were used as outgroup. Branches were colored according to the host subfamily: Aleyrodinae in blue and Aleurodicinae in red.

*Portiera* strain divergences were estimated using the host fossil records and *H. elongata* and *C. salexigens* as outgroups (fig. 3). The calibration point was set as an uniform distribution with an upper bound of 135 Ma and a lower bound of 125 Ma, based on the reports of the oldest Aleyrodinae (*Baetylus kahramanus*) and Aleurodicinae (*Gapenus rhinariatus*) fossils at the Early Cretaceous (approximately 135–125 Ma) (Drohojowska and Szwedo 2011, 2013).

Two data sets of approximately 14,000 bp each (run A and B) were used for dating *Portiera* strain divergence (table 3). BEAST2 Highest Posterior Density (HPD) obtained from both data sets for each estimated node overlapped, meaning that they were from the same distribution, and allowing the combination of both data sets in the same analyses to estimate the average parameters (run AB) (table 3). To confirm these results, PhyloBayes3 was used, obtaining results that overlapped with those of BEAST2 HPD. The estimated divergence of the two *Portiera* strains from *Aleurodicus* (*A. dispersus* and *A. floccissimus*) was 18.35 Ma (node A in fig. 3 and table 3), whereas the separation between *Portiera* strains from *T. vaporariorum* and *B. tabaci* was 90.1 Ma (node B). The divergence between *Portiera* strains from *B. tabaci* B (MEAM1 sp.) and Q (MED sp.) biotypes is much more recent: 380,000 years ago (node C). If PhyloBayes3 results are taken into account, it is possible that the divergence between B (MEAM1) and Q (MED) biotypes occurred even more recently.

**Table 3:** Divergence Dates (Myr) for the Different Nodes of *Portiera* Phylogeny (fig. 3)

Node	Description	Software	Run	Mean Age	GM Age	Median	Inf. 95% HPD	Sup. 95% HPD
Calibrator	Aleyrodidae Aleyrodinae –Aleurodicinae	BEAST2	A	129.67	125.00	134.39	129.64	129.50
			B	129.67	129.64	129.50	125.004	134.404
			**AB**	**129.47**	**129.44**	**129.22**	**125.00**	**134.31**
		PhyloBayes3	A	108.87			73.54	124.60
			B	109.41			76.07	124.51

A	Aleurodicinae *Aleurodicus dispersus–Aleurodicus floccissimus*	BEAST2	A	20.30	19.57	19.67	10.43	31.52
			B	17.68	17.14	17.16	9.62	26.71
			**AB**	**18.35**	**18.07**	**18.10**	**12.30**	**24.88**
		PhyloBayes3	A	30.97			14.83	55.19
			B	28.80			14.19	50.31

B	Aleyrodinae *Trialeurodes vaporariorum–Bemisia tabaci*	BEAST2	A	84.58	83.81	84.90	62.52	106.18
			B	93.54	92.86	94.02	71.81	114.44
			**AB**	**90.10**	**89.73**	**90.19**	**74.20**	**105.72**
		PhyloBayes3	A	63.80			40.91	84.91
			B	71.84			46.71	92.90

C	*B. tabaci* B(MEAM1)–Q(MED)	BEAST2	A	0.49	0.44	0.45	0.14	0.91
			B	0.35	0.31	0.32	0.07	0.69
			**AB**	**0.38**	**0.36**	**0.36**	**0.16**	**0.63**
		PhyloBayes3	A	0.10			0.04	0.19
			B	0.07			0.02	0.15

D	*Halomonas elongata* –*Chromohalobacter salexigens*	BEAST2	A	114.81	110.73	111.36	58.99	177.02
			B	93.54	92.86	94.02	71.81	114.44
			**AB**	**133.71**	**131.54**	**132.01**	**88.18**	**181.17**
		PhyloBayes3	A	76.55			27.25	213.38
			B	130.88			38.94	396.41

Note.—Run AB is shown in bold.

**Fig. 4.—:**
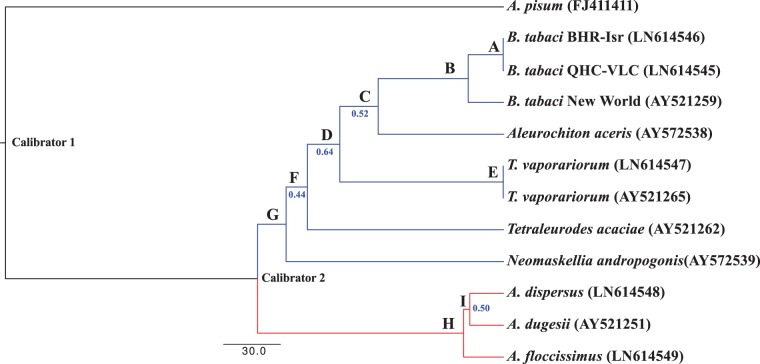
BEAST2 Bayesian inferred tree of different whiteflies. Nodes are denoted by a bold uppercase letter (see table 4). Each species is displayed with its accession number in brackets. Posterior probabilities below 1 are displayed in blue. Branch lengths are displayed in Myr. *Acyrthosiphon pisum* was used as outgroup. Branches were colored according to the subfamily: Aleyrodinae in blue and Aleurodicinae in red.

To corroborate the *Portiera* dating results, the divergence among a large number of whiteflies was estimated using the mitochondrial *COI* gene (1,341 bp). The species included and their phylogenetic relationships are shown in the fixed tree from figure 4. Again, BEAST2 and PhyloBayes3 HPDs overlapped indicating the robustness of the obtained estimates (table 4). In this case, *A. pisum* was selected as the outgroup for rooting the tree. Calibration points were set to a uniform distribution using different estimations of the emergence of the Sternorrhyncha suborder (278–250 Ma) (Wootton 1981; Shcherbakov 2000; Grimaldi and Engel 2005) and the divergence of the Aleyrodinae and Aleurodicinae subfamilies (135–125 Ma). The equivalent nodes to those of the previous *Portiera* analyses gave very similar results. The estimated divergence with BEAST2 between *A. floccissimus* and *A. dispersus* was 20.25 Ma (node H in fig. 4 and table 4), the separation of *Trialeurodes* and *Bemisia* lineages was 86.07 Ma (node D), and the divergence between the *B. tabaci* B (MEAM1) and Q (MED) biotypes was 0.21 Ma (node A). In addition, node B gave interesting information, placing the divergence of the *B. tabaci* complex in 18.43 Ma.

**Table 4:** Divergence Dates (Myr) for the Different Nodes of Whiteflies Phylogeny (fig. 4)

Node	Description	Software	Mean Age	GM Age	Median	Inf. 95% HPD	Sup. 95% HPD
Calibrator 1	Sternorrhyncha	BEAST2	263.24	263.10	262.40	250.00	277.66
		PhyloBayes3	207.66			147.12	283.65
Calibrator 2	Aleyrodidae Aleyrodinae—Aleurodicinae	BEAST2	129.74	129.71	129.60	125.00	134.42
		PhyloBayes3	130.50			125.34	134.83
A	*Bemisia tabaci* B(MEAM1)–Q(MED)	BEAST2	0.21	0.16	0.14	0.03	0.55
		PhyloBayes3	1.17			0.44	2.87
B	*B. tabaci* B(MEAM1)/Q(MED)–New World	BEAST2	18.43	17.80	17.73	9.85	28.50
		PhyloBayes3	19.87			11.16	32.44
C	*Aleurochiton aceris–Bemisia*	BEAST2	66.05	65.15	65.63	45.15	87.41
		PhyloBayes3	61.39			41.44	83.16
D	*Trialeurodes–Bemisia*/*A. aceris*	BEAST2	86.07	85.28	85.95	63.80	108.73
		PhyloBayes3	81.94			59.94	103.43
E	*Trialeurodes vaporariorum*	BEAST2	0.02	0.01	0.01	0.00	0.06
		PhyloBayes3	0.12			0.01	0.41
F	*Tetraleurodes acaciae*–{*Trialeurodes*/*Bemisia*/*A. aceris*}	BEAST2	103.09	102.46	103.53	81.23	125.08
		PhyloBayes3	95.38			73.06	116.14
G	*Neomaskellia andropogonis*–other Aleyrodinae	BEAST2	114.39	113.93	115.54	94.94	132.21
		PhyloBayes3	113.17			91.49	130.08
H	*Aleurodicus*	BEAST2	20.25	18.52	17.26	8.27	37.31
		PhyloBayes3	47.94			26.14	78.68
I	*Aleurodicus dispersus–Aleurodicus dugesii*	BEAST2	17.11	15.60	14.71	6.67	32.09
		PhyloBayes3	38.80			24.48	65.82

### Rates of Nucleotide Substitution in *Portiera* Lineages

The numbers of synonymous (d*S*) and nonsynonymous (d*N*) substitutions per site were estimated in the lineages leading to *Portiera* BT-QVLC and TV-BCN (after their divergence) and in the lineages of *Portiera* AD-CAI and AF-CAI (after their divergence). These values were divided by the mean ages of the divergence times obtained in the run AB (90.1 and 18.35 Myr, respectively), to obtain the rates of nucleotide substitution per site per year.

**Fig. 5.—:**
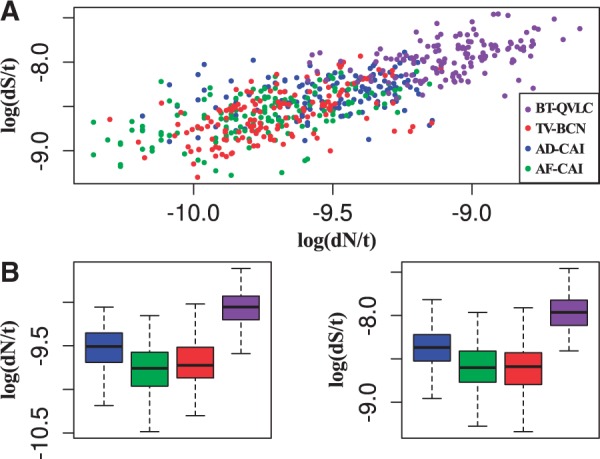
(*A*) Scatter plot of the cleaned data output from codeML. Each dot compares the logarithms of the rates of nonsynonymous and synonymous substitutions per site per year in the same lineage. (*B*) Box plot of the cleaned data. Whiskers represent the 0% and 100% quartile. Colors representing each data are the same as panel (*A*).

When the raw data were plotted (240 genes), two main clusters were observed for most of the core genes (supplementary fig. S4*A*, Supplementary Material online). *Portiera* BT-QVLC was the one with the highest rates of synonymous and nonsynonymous substitution, whereas TV-BCN, AD-CAI, and AF-CAI formed a second cluster with a lower rate. Previously to statistical tests, and as a result of exploratory data analysis step (based on descriptive statistics, histograms, density and box plots, etc.), a quality trimming of the data was performed and a 60% of the original data was kept (146 genes out of 240) (supplementary fig. S4*B*, Supplementary Material online). After trimming, three clusters were observed: *Portiera* BT-QVLC, AD-CAI, and TV-BCN/AF-CAI (fig. 5*A*).

To determine whether the rates of nonsynonymous substitution were significantly different among lineages, a Kruskal–Wallis test was performed. *Portiera* BT-QVLC was not included because it failed to pass Levene’s test when it was compared with the other *Portiera* lineages, leading to consider that the distribution of this *Portiera* is clearly different from the others. When the remaining *Portiera* strains were compared, the test gave a significant result (*P* = $9\times 10^{-12}$), supporting that not all the value distributions were equal (fig. 5*B*). Post hoc Kruskal–Wallis test confirmed that there is statistical significance to assume that AD-CAI presents a different distribution compared with AF-CAI or TV-BCN (*P* values, $4.4\times 10^{-14}$ and $4.0\times 10^{-08}$, respectively) and no significant differences between AF-CAI/TV-BCN (*P* = 0.039).

For the rates of synonymous substitution, *Portiera* BT-QVLC also failed to pass Levene’s test. Similar results for the distribution of the rates of synonymous substitution were found when the other *Portiera* lineages were compared. Comparisons of AD-CAI to AF-CAI or AD-CAI to TV-BCN (*t* test or Welch’s procedure for unequal variances *P* values: $2.2\times 10^{-13}$ and $9.34\times 10^{-12}$, respectively) supported that AD-CAI has a statistically different mean rate. In contrast, AF-CAI and TV-BCN showed no differences at mean rates (*t*-test with equal variance *P* = 0.859).

In addition, d*N*/d*S* (*ω*) were calculated for the orthologous CDS. Those with d*S* values equal to zero or *ω* values greater than 10 were trimmed, leaving 185 CDS at the final step (out of 240). *ω* values of each population followed a nonnormal distribution with equal variances. The median *ω* values for BT-QVLC, TV-BCN, AD-CAI, and AF-BCN were 0.0743, 0.0735, 0.0643, and 0.0656, respectively. When a Kruskal–Wallis test was applied, no significant differences were detected in *ω* distributions (*P* = 0.217).

Finally, the rates of substitution at genomic scale were calculated for each *Portiera* lineage (supplementary table S3 and fig. S5, Supplementary Material online). For nonsynonymous, *Portiera* BT-QVLC was close to $1.0\times 10^{-09}$, whereas the other three lineages were in the range of $2.0-3.0\times 10^{-10}$. In the case of synonymous (a rate very close to that of nonfunctional sequences), *Portiera* BT-QVLC was close to $1.0\times 10^{-08}$, whereas the other three lineages were in the range of $3.0-5.0\times 10^{-09}$. On the other hand, to determine the contribution of insect population factors to the among-lineage differences observed in *Portiera*, we determined the d*N*/*t* and d*S*/*t* rates in the four insect lineages for the mitochondrial *COI* gene and compared them with those previously estimated at genomic scale for *Portiera* (supplementary fig. S5, Supplementary Material online). Although *B. tabaci* lineage was the fast evolving for both rates, their differences with some of the other lineages were small if they are compared with those observed in *Portiera*.

## Discussion

### The Extremely Reduced and Stable Genome of *Portiera* in Most but not All Whiteflies Lineages

The comparison between *Portiera* genomes from the distant whiteflies subfamilies Aleurodicinae and Aleyrodinae has shown that after the start of the relationship of the endosymbiont and the ancestor of whiteflies, a process of genome reduction took place, which produced a small and stable genome with a gene repertoire of 319 genes (280 coding genes). As shown by the comparative analyses, this genome was maintained almost completely stable regarding gene order and gene content during the last approximately 130 Myr, except for the endosymbionts of *B. tabaci* lineage, which have experienced extensive genome rearrangements and gene losses (Sloan and Moran 2012a, 2013). The loss of a clear GC skew pattern in *Portiera* from *B. tabaci* Q (MED) and B (MEAM1) biotypes, the proliferation of short tandem repeats in intergenic regions, and the presence of large intergenic regions (also observed in *Portiera* from the New World *B. tabaci* species [AY268081]), is indicative that in the *B. tabaci* complex lineage, *Portiera* has experienced several rearrangements since its divergence from *Trialeurodes* (Baumann 2005; Santos-Garcia et al. 2012; Sloan and Moran 2012, 2013). The remaining analyzed *Portiera* lineages have retained the original gene order and GC skew. *Portiera* strains from the subfamily Aleurodicinae almost reproduce the ancestral *Portiera* state, although some genes may have been lost through convergent losses as observed in *B. aphidicola* (Gómez-Valero et al. 2004). The close phylogenetic relationship between the endosymbionts of whiteflies, psyllids and moss bugs (Thao and Baumann 2004; Kuechler et al. 2013) and the phylogenomic reconstruction of this Halomonadaceae clade, after the genome sequencing of the three endosymbionts, has led to the suggestion that the most plausible scenario was an ancestral infection of a Psyllinea (approximately 200 Ma) followed by the divergence of the Aleyrodoidae and Psylloidae clades (Santos-Garcia, Latorre et al. 2014). The genome reduction of both endosymbionts was so significant that all *Portiera* or *Carsonella* endosymbionts have lost essential genes required for the processes of genetic information transfer (Santos-Garcia et al. 2012; Sloan and Moran 2012b, 2013). The way in which these endosymbionts cope with the loss of the encoded proteins (DNA polymerase subunits, aminoacyl tRNA synthetases, etc.) may be explained by several mechanisms, for instance, the import of nuclear encoded proteins. These essential proteins may derive from bacterial horizontal gene transfer events now integrated as host’s nuclear genes (Sloan et al. 2014), or endosymbionts could be importing the same proteins as the mitochondria, as suggested recently (Santos-Garcia, Latorre et al. 2014). Although the “symbionelle” term (Reyes-Prieto et al. 2014) seems to reinforce the idea that the evolutionary history of organelles and endosymbionts has occurred in different contexts (at unicellular and multicellular organisms, respectively), it should be revisited taking into account the loss of essential genetic information transfer genes rather than a threshold in the number of genes. Finally, the recent discovery that an aphid nuclear encoded protein was transferred into *B. aphidicola* (Nakabachi et al. 2014) suggests protein import as one relevant mechanism by which endosymbionts with extremely reduced genomes may complement some of their functional deficiencies. We propose that symbionts that require the import of host proteins to fulfill their basic genetic information transfer metabolism have crossed the boundary between organelle and symbiont, and they may be no longer considered as bacterial endosymbionts.

### Complementation of Whitefly Unbalanced Diets

Ancestral whiteflies were also sap-feeders that probably lived in gymnosperm forests during the Late Jurassic and Early Cretaceous. During the Middle Cretaceous, they diversified in association with the expansion of angiosperms (Drohojowska and Szwedo 2015). Saps from both plant types are unbalanced diets that ought to be complemented by endosymbionts. The stable association of *Portiera* with whiteflies allowed the input of many amino acids and other compounds, such as carotenoids, in the appropriate concentrations. However, when *Portiera* amino acid biosynthetic pathways are observed, most are incomplete. This could suggest that they are not functional. However, because the retention of useless genes in bacterial endosymbionts is very improbable, the most plausible explanation is that at least *Portiera* strains AF-CAI and AD-CAI may synthesize, or participate in the synthesis, of the ten essential amino acids plus glycine. In contrast, BT-QVLC and TV-BCN only participate in the synthesis of eight and nine essential amino acids, respectively. The sharing of essential amino acid biosynthetic pathways was already suggested in *B. aphidicola* and the aphid *A. pisum* as a way to enable the aphid to control amino acid supply to the endosymbiont cells (Wilson et al. 2010). Shared biosynthetic pathways were also detected between *Carsonella* and the psyllid *Pachypsylla venusta* with host genes of either bacterial or eukaryotic origin (Sloan et al. 2014). In the case of the whitefly *B. tabaci*, it is possible to detect hits of missing *Portiera* genes by TBLASTN using *A. pisum* proteins against the transcriptome sequences of *B. tabaci* deposited in the NCBI database (Wang et al. 2010; Xie et al. 2012; Ye et al. 2014). For example, the *ilvE* ortholog from *A. pisum* (*ACYPI008372*) corresponds to the HP822659 and HP659950 *B. tabaci* transcripts, or the aspartate transaminase from *A. pisum* (*ACYPI000044*, *ACYP006213*, *ACYPI003009*, *ACYPI004243*) that substitutes the *aspC*/*hisC* corresponds to HP663128 and EZ958734 transcripts from *B. tabaci* (data not shown).

In contrast to the strong amino acid biosynthetic machinery, the capabilities of *Portiera* strains regarding vitamins/cofactors are scarce. As in other Sternorrhyncha, facultative endosymbionts that share the bacteriocytes with *Portiera* are probably in charge of the vitamin/cofactor production. In this case, the special endosymbiont transmission mechanism in whiteflies, where the whole bacteriocyte migrates into the oocyte (Szklarzewicz and Moskal 2001; Coombs et al. 2007; Santos-Garcia, Silva et al. 2014), could be an adaptation to ensure the whole endosymbiotic community transmission to the offspring.

It is noteworthy mentioning that all *Portiera* strains are able to produce different carotenoid conformations using the geranylgeranyl diphosphate produced by the host. Although the canonical antioxidant function of carotenoids is well known, it is possible that they are also related to an alternative source of reductive power for the endosymbiont and the host (Valmalette et al. 2012). Carotenoid biosynthetic genes were not detected in the genomes of the endosymbionts of aphids but, on the contrary, their function was substituted by several nuclear aphid genes. Apparently, a horizontal gene transfer event of fungal origin in the ancestor of aphids and adelgids was followed by their diversification through repeated series of duplication and selection (Moran and Jarvik 2010; Nováková and Moran 2012). Beta-zeacarotene is one of the two carotenoids detected in *B. tabaci* (Nováková and Moran 2012) and it is produced by *Portiera*, suggesting the idea that *Portiera* is able to export its carotenoids to the host. Although carotenoids were also detected in the psyllid *Pachypsylla venusta* (Nováková and Moran 2012), no biosynthetic genes have been detected in the genomes of the primary endosymbiont *Carsonella* or the host *P. venusta*. Thus, the origin of this compound in psyllids is unexplained except for the Asian citrus psyllid *Diaphorina citri*, in which they are synthesized by the coprimary endosymbiont *Ca. Profftella armatura*, which harbors these genes in a plasmid (Nakabachi et al. 2013).

### Dating Insect Divergence Using DNA of Obligate Endosymbionts and Mitochondria

In many evolutionary studies, it is often advantageous to have an estimate of the timescale. The use of the molecular clock with DNA, RNA, or protein sequences has started to become a frequent approach. In insects, both nuclear and/or mitochondrial DNA sequences have been used for the estimation of divergence times and rates of sequence evolution (Ho and Lo 2013). We have used sequence data from an obligate symbiont to track the divergence times of both the insects and their endosymbionts, based on the knowledge that for several endosymbiont lineages of insects there is a strict vertical transmission of the bacterial symbionts and, thus, host and endosymbiont coevolve (Baumann 2005). This is the case of the lineages of *Portiera* and whiteflies (Thao and Baumann 2004). Different works have tried to date divergence times in insect endosymbionts and their hosts based on *16S* rRNA gene divergence and the fossil record (Moran et al. 1993; Ochman et al. 1999), or more recently applying an ML approach to a wide range of genes (Patiño Navarrete et al. 2013). To our knowledge, this is the first time that DNA from an obligate mutualistic bacterium together with its host mitochondrial DNA has been used to estimate divergence times using a Bayesian approach. However, divergence time comparisons between coevolving hosts and pathogens based on Bayesian approaches have been performed with several systems, such as *Mycobacterium tuberculosis* (Comas et al. 2013) or Felidae and papillomavirus (Rector et al. 2007).

The availability of complete genomes of *Portiera* belonging to different whiteflies species has allowed the use of a large sequence data set (approximately 27 kb in this study) for divergence dating. In addition, to validate these results, the analysis was extended to a 1,341-bp mitochondrial *COI* gene alignment from a larger set of whiteflies species. Based on our analyses, the split of the lineages leading to the genera *Trialeurodes* and *Bemisia* took place during the Late Cretaceous (100.5–66.0 Ma, 95% HPD). During this period, angiosperm lineages, and probably their feeder insects, started to diverge (Drohojowska and Szwedo 2015). The origin of the genus *Bemisia* occurred later, although, due to our limited number of taxa, we can only indicate that it took place after the divergence of the genus *Aleurochiton* and *Bemisia* (87.41–45.15 Ma). The divergence of the *B. tabaci* complex was tracked by the separation of the New World and B(MEAM1)/Q(MED) species (28.5–9.85), which is considered the origin of *B. tabaci* complex (De Barro et al. 2011). This period overlaps with the spread of open communities dominated by grasses and dicotyledon herbs (Janis et al. 2002; Drohojowska and Szwedo 2015). The divergence of the two species of the genus *Aleurodicus* (24.88–12.30) also overlaps with this period. Finally, the divergence of *B. tabaci* B (MEAM1) and Q (MED) biotypes was estimated with runAB and BEAST2 in 0.63–0.16 Ma. This value was even smaller with mitochondrial *COI* and BEAST2 (0.55–0.03). Although PhyloBayes3 results from mitochondrial *COI* gave a broader divergence range for B (MEAM1) and Q (MED) (2.88–0.44), our data do not support a previous estimation reported in Boykin et al. (2013). This work estimated the divergence of the genus *Bemisia* using an approximately 600-bp mitochondrial *COI* alignment, that placed the divergence of *B. tabaci* B (MEAM1) and Q (MED) in 13 Myr (25–8) and the divergence of New World and B(MEAM1)/Q(MED) species in 48 Myr (80–34) (Boykin et al. 2013). These larger values could be attributed to the short length of the sequence alignment, to the saturation of the phylogenetic signal, to the presence of paraphyletic groups in the inferred host phylogenetic tree, and/or to the use of a speciation model not recommended with intraspecific data (more than one individual per species) (Ho et al. 2005; Drummond et al. 2006; Heled and Drummond 2012).

Finally, it seems that divergence between B (MEAM1) and Q (MED) biotypes is very recent to consider these two biotypes as different species. Moreover, it is possible that they could be at the beginning of the speciation process. Both biotypes are able to mate and produce hybrids but these hybrids seem to have viability/infertility problems indicating some reproductive barriers (reviewed in Liu et al. 2012). Moreover, the MS (Indian Ocean) biotype (shares the same common ancestor than the B and Q biotypes) is able to produce fertile hybrids when is crossed with the B (MEAM1) biotype (Thierry et al. 2011). Because the MS (Indian Ocean) should have diverge also very recently, this suggests that the species concept is still under controversy in *B. tabaci*. A major problem in cross experiments in *B. tabaci* is that few attention has been focused on analyzing the endosymbiotic communities that biotypes can harbor, most of them reported as reproductive manipulators (*Rickettsia* sp., *Wolbachia* sp., *Cardinium* sp., and *Arsenophonus* sp.). Also, it is important to take in mind that even closely related strains of the same endosymbiont can produce postzygotic reproductive barriers and start the speciation process (Brucker and Bordenstein 2012).

### Different Rates of Molecular Evolution among Lineages

Accelerated sequence evolution was early discovered as one of the main characteristics of the evolution of both coding and noncoding genes in endosymbiotic bacteria (Moran 1996). When comparing coding genes of free-living and endosymbiotic bacteria, the rates of both nonsynonymous and synonymous substitutions were higher in endosymbionts. However, the increase in the former was much higher than in the latter (Clark et al. 1999; Tamas et al. 2002). The causes for these variations include enhanced mutation rates, relaxation of purifying selection, and the effect of the random genetic drift in small populations with continuous bottlenecks, no recombination and lack of horizontal gene transfer. In the analysis of the four *Portiera* lineages, significant differences were observed among most of the lineages for both nonsynonymous and synonymous substitution rates but these two parameters were correlated (fig. 5), and also similar *ω* values were observed in the four lineages (approximately 0.06–0.07). Because synonymous changes are considered neutral or almost neutral, especially in endosymbionts where only a weak residual codon bias among high and low expressed genes is detected, we discard changes in the pressure of natural selection and effective population size and points to among-lineage differences in the rates of mutation and/or in the generation time to explain the differences in rates of substitution. The loss of *dnaQ* (DNA polymerase III subunit epsilon) and other functionally related genes in *Portiera* from *B. tabaci* (Santos-Garcia et al. 2012; Sloan and Moran 2012a, 2013) have been suggested as the reason for the observed increases in nucleotide substitution rates (Sloan and Moran 2013). These gene losses would increase the mutation rate leading to a parallel increase of both rates. However, it would not explain the small but significant difference between *Portiera* AD-CAI and the two other lineages, because they have almost identical gene repertoires. These latter differences could be explained by variations in the average generation times in the endosymbionts of each lineage. In fact, the observation of negative correlations for both mitochondrial nonsynonymous and synonymous substitution rates against generation times in invertebrates has been reported (Thomas et al. 2010).

The availability of complete *Portiera* genomes has allowed the estimation of the rates of substitution at the genomic level in the four lineages. In a broad sense, the values of *Portiera* from *B. tabaci* lineage were 3- to 4-fold higher than in any of the other *Portiera* lineages. Because of the long periods of evolution used for our estimations (more than 10 Myr), they are not affected by the known time-dependent effect, which increases the rates over short time frames due to the inclusion of the transient deleterious mutations that have not yet been removed by purifying selection and other causes (Ho and Lo 2013).

The information about molecular evolutionary rates in bacteria is scarce in the literature due to the difficulties to estimate the times of divergence without a fossil record. The comparison of the rates of nonsynonymous substitution among bacteria (free-living or endosymbiont) strongly depends on the compared genes, because natural selection acts with different strength depending on the genes and the bacterial way of life. In a study involving approximately 20 coding genes, the nonsynonymous rates for *B. aphidicola* (within subfamilies Aphidinae and Pemphiginae) and *Escherichia coli*–*Salmonella typhimurium* were estimated in $1-2\times 10^{-09}$ and $1-2\times 10^{-10}$, respectively (Clark et al. 1999). Comparing these values with those obtained in *Portiera* (supplementary table S3, Supplementary Material online), we observe that the value of *Portiera* from *B. tabaci* was closer to that of *B. aphidicola*, whereas those of the other *Portiera* lineages approach to those of free-living bacteria.

Much more analyses were reported for the rate of synonymous substitution, and for the rates in similarly evolving sequences such as pseudogenes or intergenic regions (table 5). Although the value of *Portiera* from *B. tabaci* was close to most of those reported for *B. aphidicola* and *Ca.* Blochmannia (the endosymbiont of carpenter ants) taxa, the values of the rest of *Portiera* lineages were intermediate between endosymbionts and free-living bacteria. These results suggest that an evolutionary mechanism is driving the differences among these bacterial symbionts. Because the DNA replication and repair gene repertoires of *Portiera* are much more reduced than those of the *Ca.* Blochmannia and *B. aphidicola* strains used in the previous studies, it seems improbable that the reason was a lower mutation rate in *Portiera*. Thus, as observed for mitochondrial DNA of invertebrates (Thomas et al. 2010), we suggest that the slow-evolving *Portiera* lineages display longer generation times than the endosymbionts of aphids or carpenter ants. With these slower rates, gene losses would require longer periods of time and the reduction of the gene repertoire would take place slowly, in spite of the long-term endosymbiotic association.

**Table 5:** d*S*/*t* (and related rates) in Bacteria

Substitution/site/year	Taxon	No. Genes	Sites	Study
$2.2\times 10^{-07}$	*Buchnera aphidicola*^a^	Genome	Intergenic plus dS	Moran et al. (2009)
$1.09\times 10^{-07}$	*Candidatus Blochmannia*	2^b^	d*S*	Degnan et al. (2004)
$1.5\times 10^{-08}$	*Candidatus Blochmannia*	16	Intergenic regions	Gómez-Valero et al. (2008)
$1.3\times 10^{-08}$	*Portiera* BT-QVLC	240	d*S*	This study
$1.2\times 10^{-08}$	*B. aphidicola*	2^b^	d*S*	Degnan et al. (2004)
$0.5-1\times 10^{-08}$	*B. aphidicola*	∼20	d*S*	Clark et al. (1999)
$4-5\times 10^{-09}$	*Escherichia coli–Salmonella typhimurium*	4^c^	d*S*	Clark et al. (1999)
$4.3\times 10^{-09}$	*B. aphidicola*	1	Pseudogene	Gómez-Valero et al. (2007)
$2-5\times 10^{-09}$	Portiera (others)	240	d*S*	This study
$1.3\times 10^{-09}$	*Escherichia coli–Salmonella typhimurium*	2^b^	d*S*	Degnan et al. (2004)

^a^Because substitution rates were estimated for divergences of less than 200 years, it may be overestimated Comas et al. (2013) and Ho and Lo (2013).

^b^The same two genes.

^c^The four genes with lower Codon Adaptation Index (CAI) out of approximately 20 analyzed for *B. aphidicola.*

The estimation of the rates of substitution in the mitochondrial *COI* gene of the four whitefly lineages revealed that, although insect demographic parameters may have some effect on the rates of *Portiera* evolution, the main factors (mutation rate or generation time) are endosymbiont specific.

In conclusion, after the initial and drastic genome reduction, the genome of *Portiera* became stable in both gene order and content in most of the lineages. Endosymbiont and mitochondrial sequences have been used for divergence dating placing the diversification of *B. tabaci* complex in more recent dates. Coding gene evolution is being comparatively slower in most of the *Portiera* lineages than in other insect endosymbionts. The similar variation in the synonymous and nonsynonymous rates argues that the evolutionary driver mechanisms involved are related to lineage characteristics such as variations in mutation rates or in generation times.

## Supplementary Material

Supplementary material and methods, figures S1–S5, and tables S1–S3 are available at *Genome Biology and Evolution* online (http://www.gbe.oxfordjournals.org/).

Supplementary Data
