# Supplementary material for: Genome Evolution in the Primary Endosymbiont of Whiteflies Sheds Light on Their Divergence
Source: Genome Biol Evol. 2015 Feb 25;7(3):873–88. doi: 10.1093/gbe/evv038 (PMC5322561; doi:10.1093/gbe/evv038)
Supplement: Supplementary Data [file supp_7_3_873__index.html]

Genome evolution in the primary endosymbiont of whiteflies sheds light on their divergence — Genome Evolution in the Primary Endosymbiont of Whiteflies Sheds Light on Their Divergence — Supplementary Data 

# Genome Evolution in the Primary Endosymbiont of Whiteflies Sheds Light on Their Divergence

## Supplementary Data

files

**Files in this Data Supplement:**

- Supplementary Data - pdf file
