# Supplementary material for: Genome Evolution in the Primary Endosymbiont of Whiteflies Sheds Light on Their Divergence
Source: Genome Biol Evol. 2015 Feb 25;7(3):873–88. doi: 10.1093/gbe/evv038 (PMC5322561; doi:10.1093/gbe/evv038)
Supplement: Supplementary Data [file supp_evv038_Supplementary_File.pdf]

Supplementary File

Supplementary Material and Methods

Genome Assembly and Annotation

The qualities of the sequences generated from three Illumina MPET libraries (one for each strain) were assessed using FastQC (version v0.10.1) (Simon, 2010). The two adapters identified as contaminants in each of the libraries were filtered using preprocess from SGA (version 0.9.37) (Simpson and Durbin, 2010). BWA's (Li and Durbin, 2009) trimming was performed on the reads using 20 as the threshold and reads smaller than 60 were discarded. Reads were indexed and assembled using the metagenome option from SGA using kmers of size 51 and, to discard reads not belonging to Portiera (species with the expected highest coverage), a minimum coverage of 200 was required for each kmer.

Using ABACAS (version 1.3.2) (Assefa et al., 2009) and Mauve (version 2.3.1) (Darling et al., 2010), the contigs matching to previously sequenced *Portiera* strains were selected and used for scaffolding using SSPACE (version 2.0) (Boetzer et al., 2011) with contig extension requiring a minimum of 50 base overlap and a coverage of 30 reads. At least 200 links and an overlap of 30 bases trimming the last 10 bases on each contig were required to join a pair of contigs. A subset of the reads from the Illumina MPET libraries and the 454 Titanium reads were mapped using Bowtie2 (version 4.5.4) (Langmead and Salzberg, 2012) and gsMapper (version 2.8) (Roche Diagnostics Corporation, 2014), to evaluate the coverage and reliability of the assemblies. These subsets were used as input for UGENE (version 1.11.5) (Okonechnikov et al., 2012) and polisher (version 1.9) (Foster et al., 2012) to identify and correct missasemblies.

Genomes were annotated using the pipeline prokka (version 1.5.2) for gram negative organisms with an e-value of $1x10^{-03}$ and manually curated. The gene *dnaK* was identified and used to set the coordinates of the genome. Inverted repeats (90% identity and larger than 50 bp) and tandem repeats (7 bp as minimum period) were screened with the Repeat Finder plugin implemented in UGENE. Genome plots were made with circos (Krzywinski et al., 2009). They are deposited at the European Nucleotide Archive (ENA) under the studies IDs PRJEB4469 (TV-BCN), PRJEB4466 (AD-CAI) and PRJEB4468 (AF-CAI).

Pathway Tools (version 18.0) (Karp et al., 2002) in combination with BioCyc/MetaCyc (Caspi et al., 2014) and BRENDA (Schomburg et al., 2014) databases were used for metabolic reconstructions and comparisons. Metabolism reconstructions are deposited at the SRI registry (SRI International, 2014). The metabolic graph was modified with Inkscape.

References

Assefa S, Keane TM, Otto TD, Newbold C, Berriman M. 2009. ABACAS: algorithm-based automatic contiguation of assembled sequences. Bioinformatics. 25(15):1968–1969.

Boetzer M, Henkel CV, Jansen HJ, Butler D, Pirovano W. 2011. Scaffolding pre-assembled contigs using SSPACE. Bioinformatics. 27(4):578–9.

Caspi R, et al. 2014. The MetaCyc database of metabolic pathways and enzymes and the BioCyc collection of Pathway/Genome Databases. Nucleic Acids Res. 42(Database issue):D459–471.

Darling AE, Mau B, Perna NT. 2010. progressiveMauve: multiple genome alignment with gene gain, loss and rearrangement. PLoS One. 5(6):e11147.

Foster B, et al. 2012. POLISHER : a tool for using ultra short reads in genome sequence improvement. `http://jgi.doe.gov/data-and-tools/polisher/`.

Karp PD, Paley S, Romero P. 2002. The Pathway Tools software. Bioinformatics. 18 Suppl 1:S225–232.

Krzywinski M, et al. 2009. Circos: an information aesthetic for comparative genomics. Genome Res. 19(9):1639–1645.

Langmead B, Salzberg SL. 2012. Fast gapped-read alignment with Bowtie 2. Nat. Methods. 9(4):357–359.

Li H, Durbin R. 2009. Fast and accurate short read alignment with Burrows-Wheeler transform. Bioinformatics. 25(14):1754–1760.

Okonechnikov K, Golosova O, Fursov M. 2012. Unipro UGENE: a unified bioinformatics toolkit. Bioinformatics. 28(8):1166–7.

Roche Diagnostics Corporation. 2014. 454 Sequencing. `http://www.454.com/products/analysis-software/`.

Schomburg I, Chang A, Schomburg D. 2014. Standardization in enzymology-Data integration in the world's enzyme information system BRENDA. Perspectives in Science. 1(16):15–23.

Simon A. 2010. Babraham Bioinformatics. `http://www.bioinformatics.babraham.ac.uk/projects/fastqc/`.

Simpson JT, Durbin R. 2010. Efficient construction of an assembly string graph using the FM-index. Bioinformatics. 26(12):i367–i373.

SRI International. 2014. SRI Registry of Pathway/Genome Databases. `http://biocyc.org/registry.html`.

Supplementary Figures

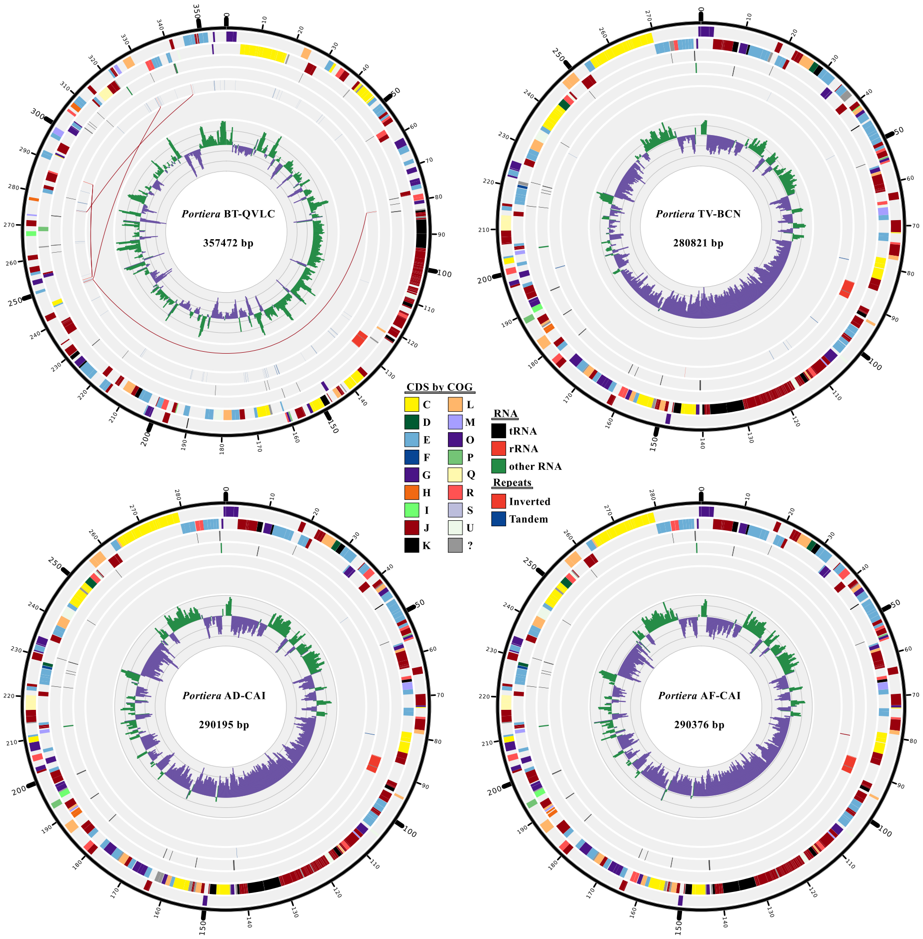

Supplementary Figure 1: Genome overview of *Portiera* strains BT-QVLC, TV-BCN, AD-CAI and AF-CAI. From inner to outer tracks: (I) Positive (green) and negative (purple) GC skew across the genome. (II) Inverted repeats (red lines and links) and Tandem repeats (blue). (III) Complementary strand noncoding RNA genes: rRNAgenes (red), transferRNA genes (black), other RNA genes (green). (IV) Direct strand noncoding RNA genes: rRNAgenes (red), transferRNAgenes (black), other RNAgenes (green). (V) Complementary strand CDS. (VI) Direct strand CDS. CDS were coloured according to their COG classification.

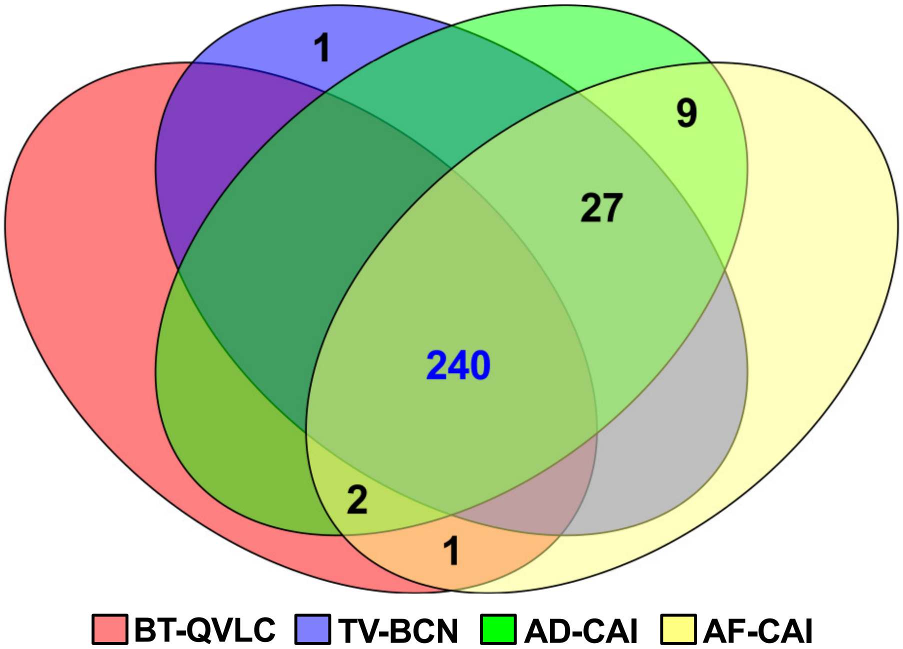

Supplementary Figure 2: Euler diagram representing the core genome, the pangenome and the strain specific coding genes. The number of genes of the core genome is highlighted in blue.

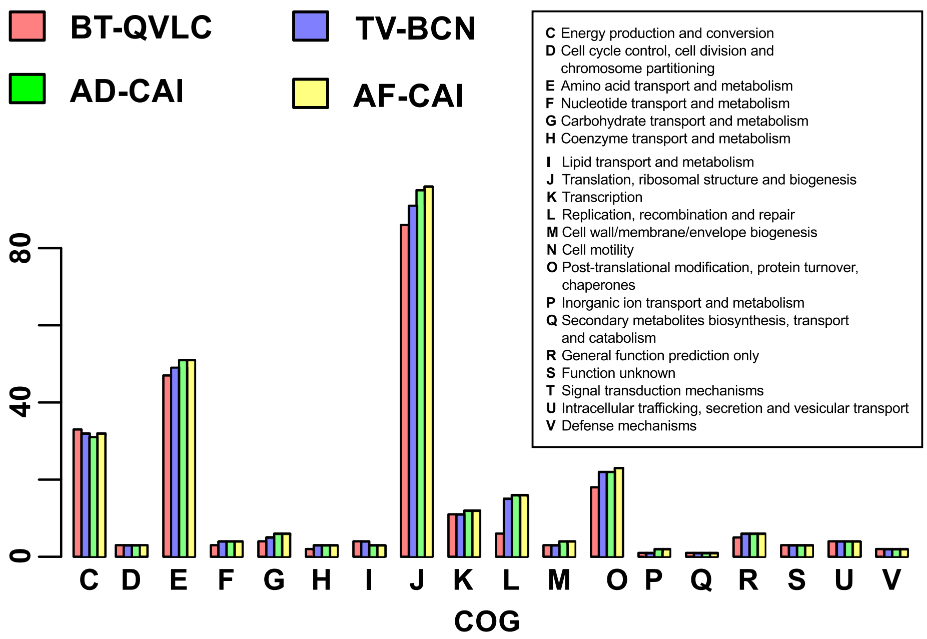

Supplementary Figure 3: Bar plot representing the number of COG hits for each *Portiera* strain proteome.

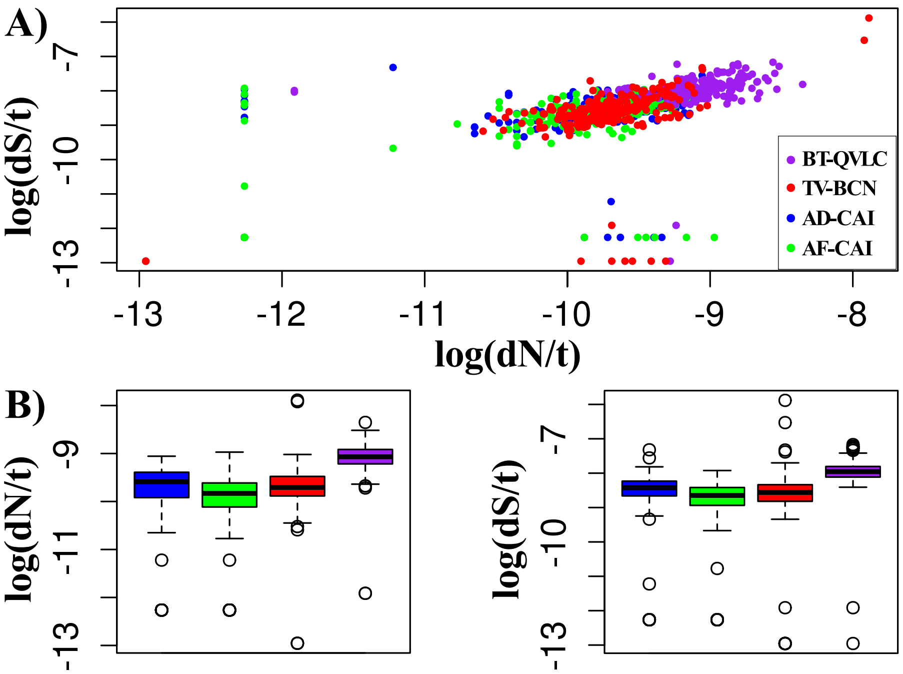

Supplementary Figure 4: **A**) Scatter plot of the raw data output from codeML. Each dot compares the logarithms of the rates of nonsynonymous and synonymous substitutios per site per year in the same lineage. **B**) Box plot of the raw date before cleaning. Whiskers represents the 0% and 100% quartile. Colors representing each data are the same as panel **A**. Notice that some dN/t and dS/t tendencies are masked by the outliers and extreme values.

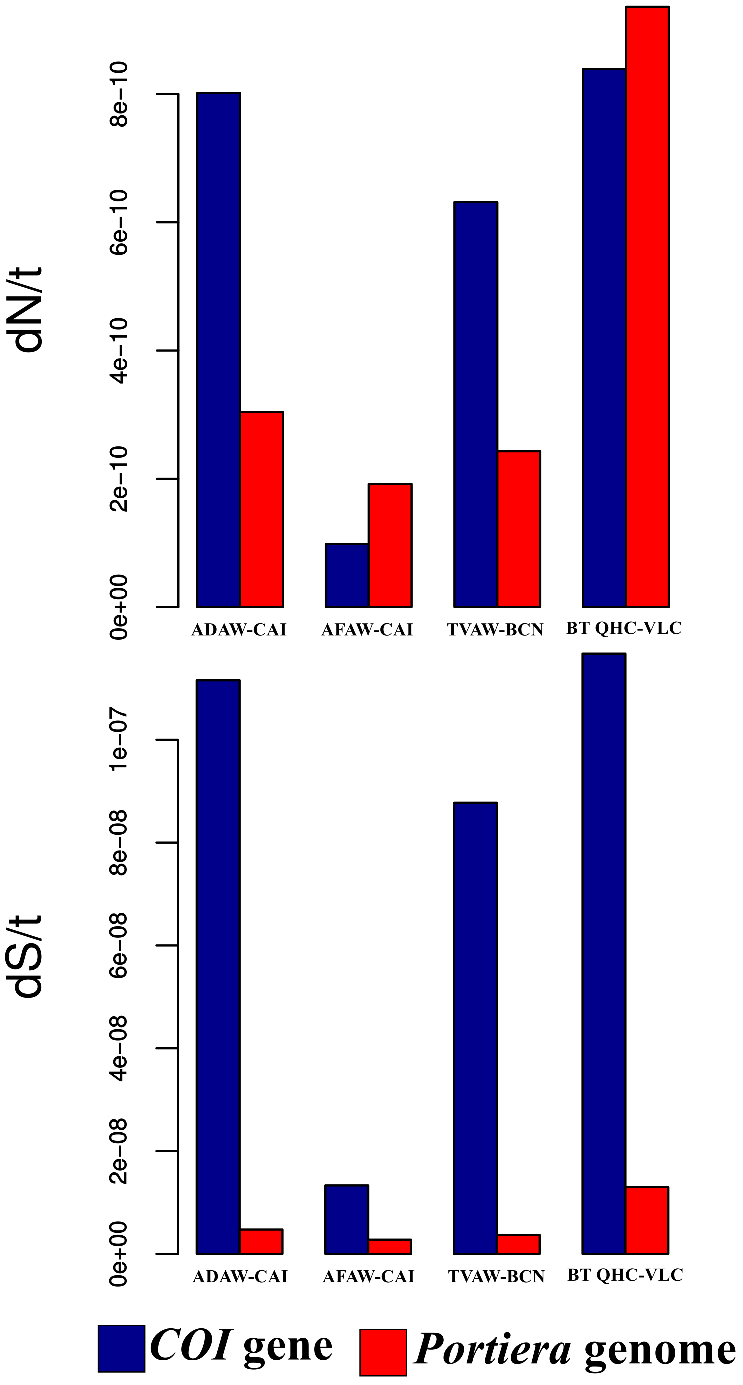

Supplementary Figure 5: Bar plots comparing dN/t (nonsynonymous substitutions per site per year) (top) and dS/t (synonymous substitutions per site per year) (bottom) between the mitochondrial *COI* gene and the genomes of *Portiera* belonging to four whitefly lineages. From left to right, *A. dispersus*, *A. floccissimus*, *T. vaporariorum*, and *B. tabaci*.

Supplementary Tables

Supplementary Table 1: Lost genes in *Portiera* from *B. tabaci versus* LCA

	KO	gene name Portiera	KEGG description	Type	Subtype	Name
1	K01358	*clpP*	ATP-dependent Clp protease, protease subunit [EC:3.4.21.92]	Cellular Processes	Cell growth and death	Cell cycle - Caulobacter
2	K03544	*clpX*	ATP-dependent Clp protease ATP-binding subunit ClpX	Cellular Processes	Cell growth and death	Cell cycle - Caulobacter
3	K03101	*lspA*	signal peptidase II [EC:3.4.23.36]	Genetic Information Processing	Folding, sorting and degradation	Protein export
4	K03100	*lepB*	signal peptidase I [EC:3.4.21.89]	Genetic Information Processing	Folding, sorting and degradation	Protein export
5	K00761	*upp*	uracil phosphoribosyltransferase [EC:2.4.2.9]	Genetic Information Processing	Folding, sorting and degradation	Pyrimidine metabolism
6	K05592	*deaD*	ATP-dependent RNA helicase DeaD [EC:3.6.4.13]	Genetic Information Processing	Folding, sorting and degradation	RNA degradation
7	K03695	*clpB*	Chaperone protein ClpB	Genetic Information Processing	Folding, sorting and degradation	
8	K02338	*dnaN*	DNA polymerase III subunit beta [EC:2.7.7.7]	Genetic Information Processing	Replication and repair	DNA replication
9	K02342	*dnaQ*	DNA polymerase III subunit epsilon [EC:2.7.7.7]	Genetic Information Processing	Replication and repair	DNA replication
10	K02343	*dnaX*	DNA polymerase III subunit gamma/tau [EC:2.7.7.7]	Genetic Information Processing	Replication and repair	DNA replication
11	K02340	*holA*	DNA polymerase III subunit delta [EC:2.7.7.7]	Genetic Information Processing	Replication and repair	DNA replication
12	K02341	*holB*	DNA polymerase III subunit delta' [EC:2.7.7.7]	Genetic Information Processing	Replication and repair	DNA replication
13	K03111	*ssb*	single-strand DNA-binding protein	Genetic Information Processing	Replication and repair	DNA replication
14	K01159	*ruvC*	crossover junction endodeoxyribonuclease RuvC [EC:3.1.22.4]	Genetic Information Processing	Replication and repair	Homologous recombination
15	K07447	*yqgF*	Putative Holliday junction resolvase	Genetic Information Processing	Replication and repair	Homologous recombination
16	K03572	*mutL*	DNA mismatch repair protein MutL	Genetic Information Processing	Replication and repair	Mismatch repair
17	K01872	*alaS*	alanyl-tRNA synthetase [EC:6.1.1.7]	Genetic Information Processing	Translation	Aminoacyl-tRNA biosynthesis
18	K01874	*metG*	methionyl-tRNA synthetase [EC:6.1.1.10]	Genetic Information Processing	Translation	Aminoacyl-tRNA biosynthesis
19	K01867	*trpS*	tryptophanyl-tRNA synthetase [EC:6.1.1.2]	Genetic Information Processing	Translation	Aminoacyl-tRNA biosynthesis
20	K02907	*rpmD*	large subunit ribosomal protein L30	Genetic Information Processing	Translation	Ribosome
21	K03595	*era*	GTPase Era	Genetic Information Processing	Translation	Ribosome
22	K02838	*frr*	Ribosome recycling factor	Genetic Information Processing	Translation	Ribosome
23	K02528	*rsmA*	Ribosomal RNA small subunit methyltransferase A	Genetic Information Processing	Translation	Ribosome
24	K03685	*rnc*	ribonuclease III [EC:3.1.26.3]	Genetic Information Processing	Translation	Ribosome biogenesis in eukaryotes
25	K08137	*galP*	Galactose transporter	Environ. Information Processing		
26	K01755	*argH*	argininosuccinate lyase [EC:4.3.2.1]	Metabolism	Global and overview maps	Biosynthesis of amino acids
27	K00215	*dapB*	4-hydroxy-tetrahydrodipicolinate reductase [EC:1.17.1.8]	Metabolism	Global and overview maps	Biosynthesis of amino acids
28	K01778	*dapF*	diaminopimelate epimerase [EC:5.1.1.7]	Metabolism	Global and overview maps	Biosynthesis of amino acids
29	K00600	*glyA*	glycine hydroxymethyltransferase [EC:2.1.2.1]	Metabolism	Global and overview maps	Biosynthesis of amino acids
30	K01586	*lysA*	diaminopimelate decarboxylase [EC:4.1.1.20]	Metabolism	Global and overview maps	Biosynthesis of amino acids
31	K00615	*tktA*	transketolase [EC:2.2.1.1]	Metabolism	Global and overview maps	Biosynthesis of amino acids
32	K00791	*miaA*	tRNA dimethylallyltransferase [EC:2.5.1.75]	Metabolism	Global and overview maps	Biosynthesis of secondary metabolites
33	K03801	*lipB*	lipoyl(octanoyl) transferase [EC:2.3.1.181]	Metabolism	Metab. of cofactors and vitamins	Lipoic acid metabolism
34	K03530	*hupB*	DNA-binding protein HU-beta			
35	K01362	*mucD*	Serine protease MucD			
36	K04774	*sohB*	Putative protease SohB			
37			hypothetical protein (ortholog of PalTV_187)			
38			putative ABC-type transporter protein (PAQ_201)			

Supplementary Table 2: Lost genes in *Portiera* from *T. vaporariourum* versus LCA

	KO	gene name Portiera	ko map	Type	Subtype	Name
1	K01872	*alaS*	alanyl-tRNA synthetase [EC:6.1.1.7]	Genetic Information Processing	Translation	Aminoacyl-tRNA biosynthesis
2	K01874	*metG*	methionyl-tRNA synthetase [EC:6.1.1.10]	Genetic Information Processing	Translation	Aminoacyl-tRNA biosynthesis
3	K02907	*rpmD*	large subunit ribosomal protein L30	Genetic Information Processing	Translation	Ribosome
4	K03685	*rnc*	ribonuclease III [EC:3.1.26.3]	Genetic Information Processing	Translation	Ribosome biogenesis in eukaryotes
5	K02863	*rplA*	large subunit ribosomal protein L1	Genetic Information Processing	Translation	Ribosome
6	K07447	*yqgF*	Putative Holliday junction resolvase	Genetic Information Processing	Replication and repair	Homologous recombination
7	K00791	*miaA*	tRNA dimethylallyltransferase [EC:2.5.1.75]	Genetic Information Processing	Translation	tRNA modification A37
8	K00600	*glyA*	glycine hydroxymethyltransferase [EC:2.1.2.1]	Metabolism	Global and overview maps	Biosynthesis of amino acids
9	K00615	*tktA*	transketolase [EC:2.2.1.1]	Metabolism	Global and overview maps	Biosynthesis of amino acids
10	K01523	*hisE*	phosphoribosyl-ATP pyrophosphohydrolase [EC:3.6.1.31]	Metabolism	Global and overview maps	Biosynthesis of amino acids
11	K03386	*ahpC*	Alkyl hydroperoxide reductase subunit C	Metabolism		peroxidasa
12	K03530	*hupB*	DNA-binding protein HU-beta			
13			putative ABC-type transporter protein (PAQ_201)			

Supplementary Table 3: Genomic dN/t and dS/t values for *Portiera* lineages

	BT-QVLC	TV-BCN	AD-CAI	AF-CAI
dN/t	$9.36x10^{-10}$	$2.43x10^{-10}$	$3.04x10^{-10}$	$1.92x10^{-10}$
dS/t	$1.3x10^{-08}$	$3.69x10^{-09}$	$4.7410x10^{-09}$	$2.77x10^{-09}$
